# Salivary immunity of elite collegiate American football players infected with SARS-CoV-2 normalizes following isolation

**DOI:** 10.1038/s41598-022-12934-7

**Published:** 2022-05-31

**Authors:** Joshua Granger, Eunhan Cho, Kevin Lindsey, Nathan Lemoine, Derek Calvert, Jack Marucci, Shelly Mullenix, Hollis O’Neal, Brian A. Irving, Neil Johannsen, Guillaume Spielmann

**Affiliations:** 1 School of Kinesiology, Louisiana State University, 91 Huey P. Long Fieldhouse, Baton Rouge, LA 70803 USA; 2 LSU Athletics, LSU, Baton Rouge, LA 70803 USA; 3 Louisiana State University Health Sciences Center, Baton Rouge, LA 70803 USA; 4 Our Lady of the Lake, Baton Rouge, LA 70810 USA; 5 Pennington Biomedical Research Center, Baton Rouge, LA 70808 USA

**Keywords:** Viral infection, Diagnostic markers

## Abstract

The impact of COVID-19 on systemic immunity in the general population has been well characterized, however the short-term effects of COVID-19 infection on innate salivary immunity in elite-level athletes are unknown. Therefore, this study aimed to determine whether elite college football athletes had altered salivary immunity following the CDC-recommended isolation post-SARS-CoV-2 infection. Salivary samples were obtained from fourteen elite football players who tested positive for SARS-CoV-2 (n = 14), immediately after CDC-recommended isolation (average days = 14 ± 2 days) and fifteen controls who remained uninfected with SARS-CoV-2. Biomarkers of innate salivary immunity (sIgA and alpha-amylase), antimicrobial proteins (AMPs, i.e., HNP1-3, lactoferrin, LL-37) and lung inflammation (SPA, SPLI, and Neutrophil Elastase-alpha-1-antitrypsin complex) were measured. Independent student t-tests were used to determine changes in biomarkers between groups. Although all AMP levels were within normal range, Human Neutrophil Defensin 1–3 concentrations and secretion rates were higher in SARS-CoV-2+ compared to SARS-CoV-2–. This suggests that the CDC-recommended isolation period is sufficient to ensure that athletes’ salivary immunity is not compromised upon return to sports, and athletes post-COVID-19 infection do not appear to be at greater risk for secondary infection than those with no history of COVID-19.

## Introduction

Severe acute respiratory syndrome coronavirus 2 (SARS-CoV-2) the causal agent of COVID-19, is transmitted by close contact with infected individuals through droplets and aerosols, and can induce severe pneumonia^1^, cytokine storm^2^ and even death^3^. Transmission of SARS-CoV-2 through these droplets and aerosols occur in activities involving the oral cavity such as breathing, coughing, and sneezing^4^. Aerosols produced by coughing or sneezing are viable for hours^5^ and enter the lungs through the mouth and nose from inhalation^6^. In addition, upper airways are also the primary route of infection for various bacteria^7^ and viruses^8^, which can lead to serious secondary infections in both COVID-19 patients^9^ and adults who recently recovered from other infections^10^. Since secondary pneumonias are associated with increased risk of death and poor health outcomes^9,11^, limiting person-person contact during the recovery period may reduce the risk of secondary infections, as person-to-person contact is the main route of transmission of respiratory pathogens.

Though the exact mechanism for the increased risk of secondary pneumonia after viral infection is unknown, disruption of the mucosal barrier and an impaired host response during the recovery period is suspected^12,13^. One of the primary sources of mucosal and salivary immunity includes antimicrobial proteins (AMPs), which are crucial in keeping immunocompetent hosts healthy from various infections^14^. These AMPs such as salivary IgA (sIgA), human neutrophil defensins (HNP1-3), lactoferrin, secretory leucocyte protease inhibitor (SLPI), and salivary surfactant protein A (SP-A) protect against bacterial, viral, and fungal infections^15–24^. sIgA and alpha-amylase (AMY) prevent bacterial and viral cells from adhering to oral surfaces^25,26^ and, in particular, prevent respiratory viruses from adhering to these surfaces^27,28^. In addition, HNPs, lactoferrin, and SLPI have broad antimicrobial activities via oral secretions^20,23^ to help maintain oral health^29,30^. Pertinent to SARS-CoV-2 systemic inflammation, these AMPs can suppress^31–34^ or balance immune inflammation in the lungs and airways^35^, a potential fatal side effect of the SARS-CoV-2 infection. Additionally, “Long COVID”^36^ is becoming a more significant concern on long-term health, possibly impacting the salivary immune system after recovery. Therefore, determining the long-lasting effects of COVID-19 on salivary immunity is essential to understanding the prevention of future respiratory infection.

Potential alterations in innate salivary immunity are particularly relevant in athletes undergoing high-intensity training, since repeated bouts of intense exercise have been purported to negatively impact immune competency^37^. While the immunosuppressive effect of high intensity exercise, and its associated increased risk of infection in athletes remains disputed^38^, studies have shown that healthy American football athletes exhibit significant decreases in sIgA concentration and secretion rates throughout their competitive season^39^. On the contrary, isolated acute bouts of high-intensity interval training are likely to exert protective effects on the salivary immune response of highly-trained men and women^40,41^, and regular periodized training does not lead to a significant change in sIgA^42^. Further, prolonged exercise does not significantly alter salivary IgA concentrations but does increase the concentration and secretion rates of other AMPs, including HNP1-3 and LL-37^43^. Thus, although recent evidence suggests that high-intensity exercise does not cause immunodepression in healthy adults, the salivary immune competency of elite level athletes who recovered from COVID-19 remains unknown, especially considering infection routes for SARS-CoV-2 are predominantly found in salivary glands^44^.

Given the importance of salivary immunity in COVID-19, specifically in athletes where close contact is unavoidable during intensive training and competition for many sports^45^, the purpose of the study is to examine markers of salivary immunity in athletes who tested positive of COVID-19. The impact of COVID-19 on the salivary immunity of elite-level athletes immediately following the CDC-recommended 10 days of isolation due to SARS-CoV-2 infection is currently unknown. As such, the goal of this study was to characterize salivary immune competency in elite-level NCAA Division I football players who had either never been infected with SARS-CoV-2, or who had recovered from COVID-19. We aim to assess recovery of the respiratory immunity in an effort to provide information for the safety of return to competition after isolation.

## Methods

### Participants

A total of twenty-nine NCAA Division I American football players provided LSU-IRB approved written and informed consent to participate in this study (mean ± S.D. age 20 ± 1.3 yrs., weight 105 ± 28 kg, height 186 ± 8 cm). Fourteen of the participants tested positive for SARS-CoV-2 and fifteen football players with no history of SARS-CoV-2 infection served as controls (Table 1). All participants were tested for SARS-CoV-2 using a nasopharyngeal-swab with samples sent to an independent clinic to detect SARS-CoV-2 by using real-time polymerase chain reaction^46^. Among the SARS-CoV-2 participants, 6 reported mild symptoms (i.e., sinus congestion, headaches, loss of taste and smell, chills, or fatigue) and 8 remained asymptomatic throughout isolation period. None of the SARS-CoV-2 negative participants reported symptoms associated with SARS-CoV-2. Following an average of 14 ± 2 days of isolation (range 10–16 days SARS-CoV-2 detection) and the team physician cleared the participants who tested positive, they reported to the laboratory for testing. Control participants reported to the laboratory without having to isolate. We performed data collection prior to the availability of SARS-CoV-2 vaccines.

**Table 1:** Participant’s physical characteristics (mean ± S.D.).

	SARS-CoV-2+	SARS-CoV-2-
n	14	15
Age	19.8 ± 0.4	20.0 ± 0.5
Height (cm)	185.1 ± 2.2	188.9 ± 2.9
Weight (kg)	102.1 ± 8.2	118.6 ± 8.2
Time post infection (days)	14 ± 2	n/a
Asymptomatic	8	n/a
Mild Symptoms	6	n/a
Severe Symptoms	0	n/a

### Saliva sampling

Saliva samples were collected using non-invasive synthetic salivates swabs (Salimetrics SOS; Carlsbad, CA). Participants were asked to rinse their mouth with water and place a previously weighed swab under their tongues for 3-min to measure salivary flow rate. Salivette weight was recorded before and after saliva collection. The difference in salivette weight was divided by the collection time to determine saliva flow rate. After collecting and weighing the swab, participants repeated the collection with a second salivette kept under their tongue until fully saturated with saliva. Salivettes were immediately centrifuged at 1500* g* for 10 min and saliva samples were stored in a -80C freezer until analysis for biomarkers of salivary immunity.

### Salivary biomarkers of immunity and lung health

Salivary immunity was evaluated using commercially available enzyme-linked immunosorbent assay (ELISA) kits. First, mucosal immune competency was characterized by measuring salivary sIgA and alpha-amylase (Salimetrics, State College, PA, USA). Next, salivary antimicrobial proteins (AMP) HNP1-3, LL-37 (Hycult Biotech, Uden, The Netherlands), and lactoferrin (Biomatik, Kitchener, Ontario, Canada) concentrations were determined. Finally, lung inflammation, a marker of overall lung health, was assessed by salivary surfactant protein A (SP-A) (Biomatik, Kitchener, Ontario, Canada), secretory leucocyte protease inhibitor (SLPI) (R&D Systems, Minneapolis, MN, USA) and salivary neutrophil Elastase-alpha-1-antitrypsin complex (Hycult Biotech, Uden, The Netherlands) concentrations^47,48^. According to manufacturers’ instructions, all samples were tested in duplicate, read on a plate reader (SprectraMax i3x, Molecular Devices; San Jose, CA) and concentrations were calculated on absorbance reading based on standard curves. Salivary biomarker concentrations were then converted to secretion rates by multiplying the concentrations by salivary flow rate. Unfortunately, technical limitations associated with saliva collection led to low volume recovery in some participants, and some analyses were conducted on a reduced sample size.

### Statistical analysis

All data were assessed for assumptions of normality using the Shapiro–Wilk test and constant error variance prior to formal statistical testing. Skewed data were normalized by logarithmic transformation. An independent student t-test was used to identify differences in participant characteristics and biomarkers of immunity and lung health between SARS-CoV-2 + and SARS-CoV-2- groups. Statistical analysis was performed with JMP Pro 15 (SAS; Cary, NC). Statistical significance was accepted at *p* < 0.05 and normally distributed data are presented as mean ± S.D. Non-normally distributed data are presented as geometric means ± S.D.

### Institutional review board

The study was conducted according to the guidelines of the Declaration of Helsinki and approved by the Institutional Review Board LSU.

### Informed consent

Informed consent was obtained from all subjects involved in the study.

## Results

### Participant characteristics

Participant characteristics are presented in Table 1. There were no differences in anthropometric characteristics between SARS-CoV-2 positive and negative participants (*p* > 0.05). Participants diagnosed with SARS-CoV-2 exhibited mild symptoms lasting 1 to 2 days with no severe symptoms reported. Participants from the control group did not report any symptoms or discomforts throughout the study.

### SARS-CoV-2 infection is not associated with chronic impairments in salivary immunity in athletes

**Figure 1:**
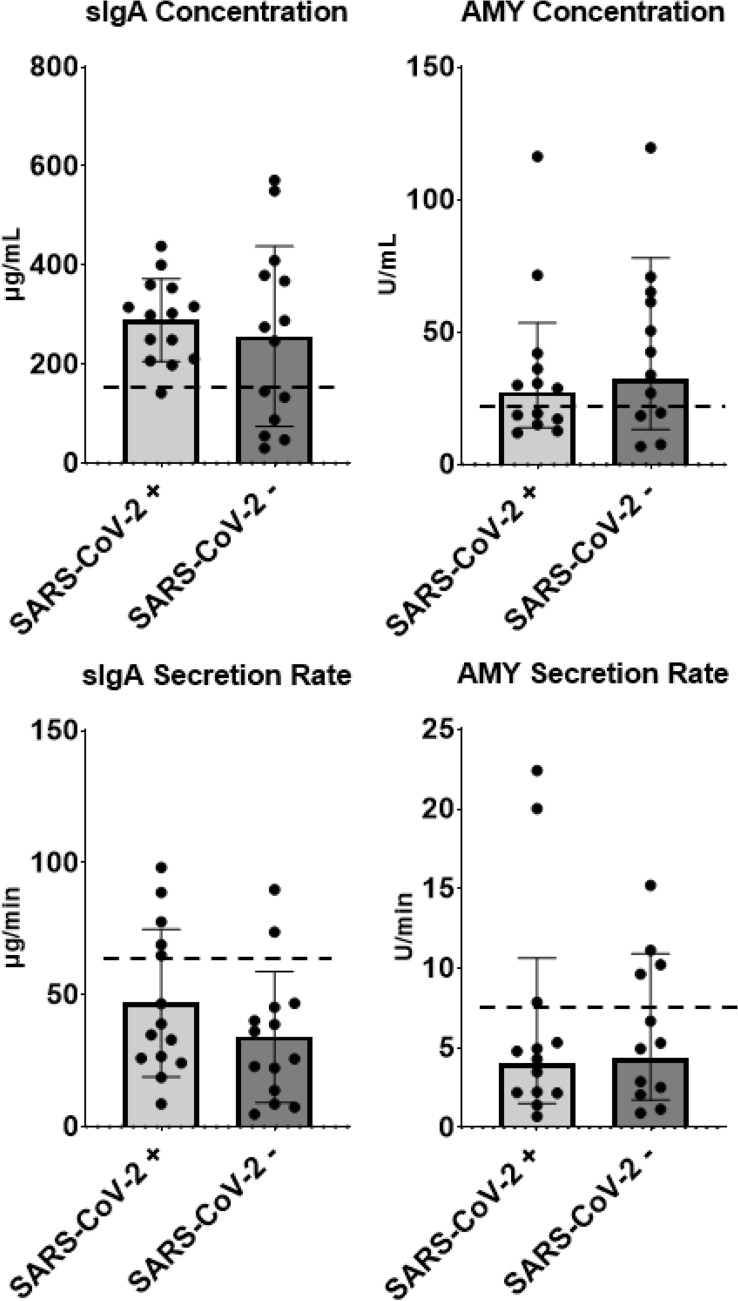
Concentrations and secretion rates of biomarkers of salivary immunity in athletes who had been infected with SARS-CoV-2 and those who had remained infection-free. No significant differences were found. Salivary IgA (SIgA), alpha-amylase (AMY), dash line (–) represents normal values reported in elite athletes. Concentrations and secretion rates for sIgA (n SARS-CoV-2 +  = 14; n SARS-CoV-2- = 14) are presented as means ± S.D while alpha-amylase (n SARS-CoV-2 +  = 13; n SARS-CoV-2- = 12) are presented as geometric means ± S.D.

No difference in salivary flow rates (0.14 ± 1.9 mL * min^−1^; 0.14 ± 1.4 mL * min^−1^ respectively; *p* = 0.99) were observed between SARS-CoV-2 + and SARS-CoV-2- groups. The salivary immune markers sIgA (289 ± 38 ug * mL^−1^; 256 ± 38 ug * mL^−1^ respectively; *p* = 0.55) and alpha-amylase (27.28 ± 2.0 U * mL^−1^; 32.1 ± 2.4 U * mL^−1^ respectively; *p* = 0.61) concentrations were not different between groups (Fig. 1). No difference was found in sIgA secretion rate (46.9 ± 7.1 ug * min^−1^; 34.0 ± 7.1 ug * min^−1^ respectively; *p* = 0.21) or AMY secretion rates (4.02 ± 2.65 U * min^−1^; 4.35 ± 2.51 U * min^−1^ respectively; *p* = 0.84) between groups (Fig. 1).

### Differences in salivary antimicrobial protein concentrations

**Figure 2:**
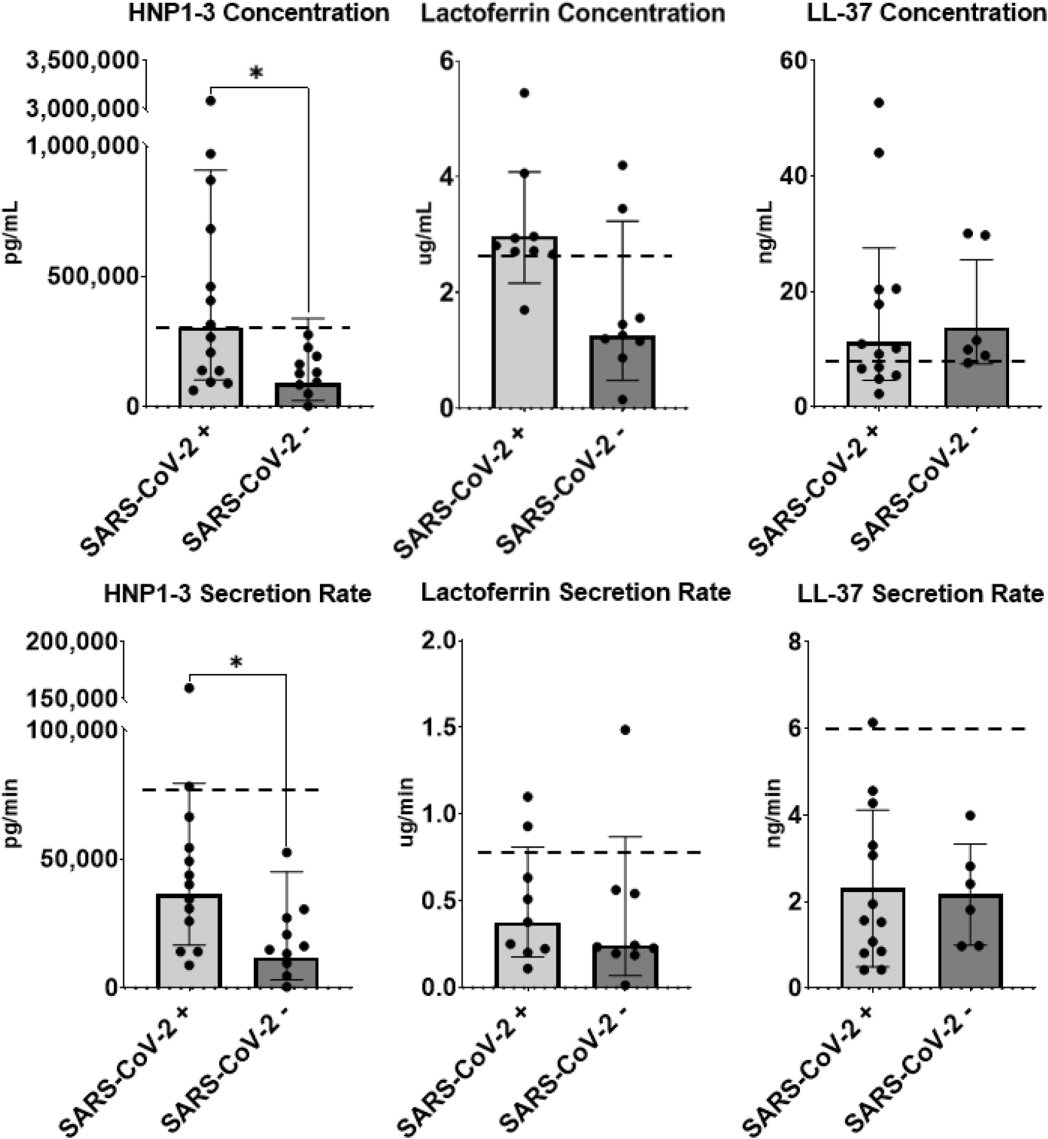
Concentrations and secretion rate of biomarkers of salivary immunity in athletes who had been infected with SARS-CoV-2 and those who had remained infection-free. Human neutrophil defensins (HNP1-3), and dash line (–) represents normal values in elite athletes. *p < 0.005. Concentrations and secretion rates for LL-37 (n SARS-CoV-2 +  = 13 ; n SARS-CoV-2- = 6) are presented as means ± S.D while HNP1-3 (n SARS-CoV-2 +  = 14; n SARS-CoV-2- = 10) and Lactoferrin (n SARS-CoV-2 +  = 9; n SARS-CoV-2- = 9) are presented as geometric means ± S.D.

HNP1-3 concentrations (SARS-CoV-2 +: 305,120 ± 2.97 pg * mL vs. SARS-CoV-2-: 90,837 ± 3.73 pg * mL; *p* = 0.02) were significantly higher in the SARS-CoV-2 positive participants then their SARS-CoV-2 negative counterparts. Similarly, HNP1-3 secretion rates were ~ 250% higher in the SARS-CoV-2 + group compared to SARS-CoV-2- groups (SARS-CoV-2 + : 36,270 ± 9,907 pg * min^−1^ vs. SARS-CoV-2-: 11,925 ± 8,689 pg * min^−1^; *p* = 0.02) (Fig. 2). The SARS-CoV-2 positive group appeared to contain one outlier for HNP1-3 concentrations and secretion rates. Since completing the statistical analysis with and without including this participant, still showed that SARS-CoV-2 + athletes had significantly greater concentrations and secretion rates of HNP1-3, the data presented includes the participant. No differences were found in other AMP concentrations between the SARS-CoV-2 + and SARS-CoV-2- groups (Fig. 2). Lactoferrin secretion rate and LL-37 secretion rates were not different between groups (0.38 ± 2.15 ug * min^−1^; 0.24 ± 3.56 ug * min^−1^; *p* = 0.39 and 2.31 ± 0.46 ng * min^−1^; 2.16 ± 0.67 ng * min^−1^; *p* = 0.86 respectively; Fig. 2).

### Biomarker of lung inflammation and health

**Figure 3:**
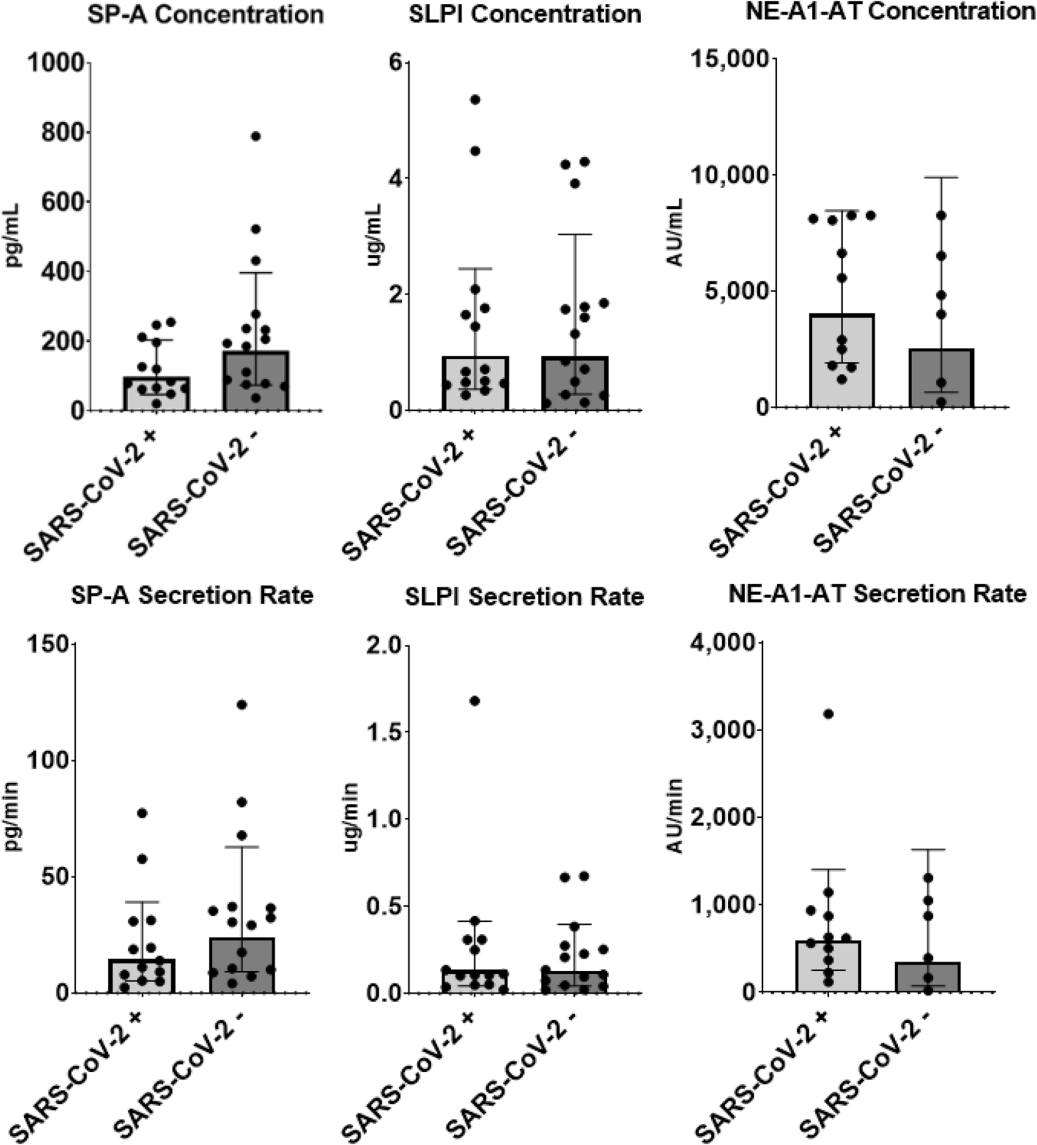
Concentrations and secretion rates of biomarkers of lung inflammation in athletes who had been infected with SARS-CoV-2 and those who had remained infection-free. No significant differences were found in secretory leucocyte protease inhibitor (SLPI), salivary surfactant protein A (SP-A), salivary neutrophil Elastase-alpha-1-antitrypsin complex (NE-A1-AT). Concentrations and secretion rates for SP-A (n SARS-CoV-2 +  = 13; n SARS-CoV-2- = 15), SLPI (n SARS-CoV-2 +  = 14; n SARS-CoV-2- = 15) and NE-A1-At (n SARS-CoV-2 +  = 11; n SARS-CoV-2- = 6) are presented as geometric means ± S.D.

A trend for greater SP-A, a marker of lung inflammation, was observed in the SARS-CoV-2- group (SARS-CoV-2−: 97.25 ± 2.09 pg * ml^−1^ vs. SARS-CoV-2 +: 170.80 ± 2.32 pg * ml^−1^; *p* = 0.07) compared to the SARS-CoV-2 + group. However, other biomarkers of lung inflammation such as SPLI (0.95 ± 2.58 ug * ml^−1^; 0.93 ± 3.27 ug * ml^−1^ respectively; *p* = 0.96), and NE-A1-AT (4029 ± 2.10 AU * ml^−1^; 2534 ± 3.91 AU * ml^−1^ respectively; *p* = 0.37) were not different between groups. Similarly, salivary secretion rates remained identical between participants who had recovered from COVID-19 and those that were never infected with SARS-CoV-2, SPA (14.33 ± 2.73 pg * min^−1^; 23.81 ± 2.63 pg * min^−1^ respectively; *p* = 0.19), SPLI (0.13 ± 3.12 ug * min^−1^; 0.13 ± 3.07 ug * min^−1^ respectively; *p* = 0.95) and NE-A1-AT (595.90 ± 2.36 AU * min^−1^; 347.70 ± 4.70 AU * min^−1^ respectively; *p* = 0.36) (Fig. 3).

## Discussion

This study aimed to determine whether innate salivary immunity was compromised immediately following CDC-recommended isolation after SARS-CoV-2 infection in elite level athletes. The primary finding of the present study was that HNP1-3 concentrations and secretion rates were significantly higher in the SARS-CoV-2 + group compared to the control group, with no differences in other biomarkers of salivary immunity between athletes who were infected with SARS-CoV-2 and those with no previous infection. These findings collectively support for the first time that an isolation period of 14 + /− 3 days is sufficient for salivary immunity and lung inflammation to normalize in elite athletes who were infected with SARS-CoV-2.

Mucosal immunity, one of the most significant components of the immune system^49^, likely plays an important role in curtailing respiratory infections. Specifically, optimal production of salivary antimicrobial proteins (AMPs) and other soluble factors such as secretory IgA (sIgA), alpha-defensins, cathelcidin (LL-37), lactoferrin, and SLPI^50^ are believed to play a preponderant role in keeping immunocompetent hosts healthy^14^. sIgA is one of the first lines of defense against pathogens and protects against bacteria^15^ and viruses^16^. Specifically, sIgA offers protection at the site of contact against bacteria^25^ and viruses^28^, especially against respiratory viral infections^27^. Exposure to physical and psychological stressors can modulate salivary sIgA concentration and secretion rates in a wide range of active populations including recreational^51,52^, operational^53,54^ and elite athletes^39^. In addition, certain viral infections lead to a transient increase in salivary sIgA, followed by a prolonged reduction in sIgA following viral clearance^55^. Here, we found no difference in salivary sIgA concentration or secretion rate between football players who were never infected with SARS-CoV-2 and those who had just recovered from COVID-19, suggesting SARS-CoV-2 + participants have normal sIgA values. Both groups had similar concentrations of sIgA normally seen in athletes^39,56^. Since players had the same training status and were exposed to an equivalent level of psychological and academic stressors, it can be argued that any change in salivary sIgA induced by SARS-CoV-2 infection did not lead to long-lasting decrements in sIgA in these elite-level athletes. sIgA secretion rate was not different between SARS-CoV-2 positive and control groups and the values that we report here are within normal range for athletes (*p* = 0.210). Interestingly, recent evidence suggested that sIgA levels remained elevated for up to 2–3 months in individuals from the general population who had recovered from SARS-CoV-2^57^. Although we were unable to collect saliva samples on the day of infection, it could be hypothesized that salivary sIgA either remained within normal range throughout the infection and isolation periods in SARS-CoV-2 positive athletes, or that salivary sIgA levels normalized more rapidly in an elite athlete population than what is observed in the general public.

Alpha-amylase, produced in the salivary glands^58^, is one of the most abundant components in saliva^59^, and it has particular importance in immunity since it has been shown to prevent adherence and growth of bacteria on oral surfaces^26^. Interestingly, AMY concentration and secretion rates are known to respond to a variety of stressors, with increased secretion observed in response to acute exercise^60^ and psychological stressors^61^ as well as chronic psychological stressors^62^. Here, we showed no difference in alpha-amylase secretion rates between athletes who tested positive for SARS-CoV-2 and those that did not. Furthermore, the values reported in this study are within normal range for healthy young adults^63^, suggesting that our study population’s salivary immunity was not detrimentally impacted and can safely return to practice/play.

Similarly, Human Neutrophil Defensins (alpha defensins, HNP) are antimicrobial peptides that play a role in the first line of defense against infections^29^ and kill a wide variety of bacteria, fungi, and some enveloped viruses^17,18^, which is of particular interest as SARS-CoV-2 is an enveloped virus^64^. Secretion rates and concentrations of HNP1-3 have been shown to be significantly higher in children with dental caries caused by bacteria compared to healthy caries-free subjects^65,66^. Acute prolonged exercise also significantly increases HNP1-3 concentrations and secretion rates^43^. HNP1-3 was found to be significantly increased in SARS-CoV-2 positive athletes compared to the control athletes, although the SARS-CoV-2 positive values remained within normal ranges seen in athletes at rest^43^. We hypothesize that this higher concentration of HNP1-3 in the SARS-CoV-2 + group is due to a delayed immune response to SARS-CoV-2, further advocating for optimal immune protection against novel infections^67^.

Another AMP of interest, LL-37, acts as an anti-inflammatory mediator by suppressing mitogen-mediated immune responses^31^ while also being able to promote inflammation in the absence of antigenic stimulation by enhancing cytokine production^68^ releasing cytokines via human airway smooth muscle cells^69^. Additionally, lactoferrin is a protein with antimicrobial, antiviral, and antifungal properties^19^, affecting the innate immune system^70^, and affecting adaptive immunity^71^. LL-37 and lactoferrin have been shown to significantly increase during infections and inflammation^72,73^. However, exposure to physical stressors such as acute prolonged exercise is associated with an increase in LL-37 secretion even in the absence of infectious agent^43^. Conversely, salivary lactoferrin appears to be suppressed by chronic exposure to exercise bouts, with non-exercisers producing twice as much salivary lactoferrin as elite level rowers throughout a competitive season^74^. Here, we report resting LL-37 and lactoferrin concentrations and secretion rates comparable to those reported elsewhere in healthy athletes^75,76^.

Surfactant proteins (SP) SP-A is an important components of host defense against respiratory pathogens^77^. SP-A defends against virial, bacterial, and fungal infections through enhancement of phagocytosis, killing through oxidative mechanisms^24^. SP-A also plays a role in immune balancing during pulmonary inflammation, and primes acquired immunity against pathogens^35^. SP-A has also been showing to mediate suppression of inflammation in the airways^33,34^. Salivary SP-A concentrations found in this study were similar to those seen in non-smoking men^78^. Secretory leukocyte protease inhibitor (SLPI) is a protein associated with the innate immune system with the main function of protecting local tissue from inflammation^32^. SLPI has bactericidal^21^, antifungal^22^, and antiviral^23^ properties, although SLPI antiviral properties seem to be limited to deterring HIV-1 transmission via oral secretions^23^. Serum SLPI concentration was shown to be increased in nasopharyngeal carcinoma patients compared to healthy controls^79^ and an increase in SPLI with individuals with Mycobacterium tuberculosis^80^. Although, SLPI is downregulated in herpes simplex virus as part of the viruses mechanism to avoid mucosal immunity^81^. This evidence along with the findings from previous studies suggest that SLPI secretion rates are unaffected by SARS-CoV-2 and that changes in concentrations of SLPI will allow secretion rates to remain unchanged. As such, it can be concluded that the studied athlete did not present significant lung inflammation following isolation.

Hyposalivation and dry mouth has been shown to be symptoms experienced by a high proportion of SARS-CoV-2 patients^82^. In this study, we did not find significant differences between athletes with and without history of SARS-CoV-2. In addition, no athlete reported experiencing extensive dry mouth as a symptom during isolation. This suggests that elite athlete’s salivation rate may be unaffected by SARS-CoV-2 or that they had fully recovered and no longer hyposalivated.

Taken together, the data presented in this study strongly advocate for preserved salivary immunity in our cohort of elite levels athletes, and that following 14 days of isolation, players who had been infected with SARS-CoV-2 are likely to be equally protected against other viral infections than those with no previous SARS-CoV-2 infection^83^. It is likely that the high level of fitness of our study population could explain the lack of severe symptoms in response to SARS-CoV-2 infection, and the rapid recovery of salivary immune health. Interestingly, high physical fitness limits viral reactivity during exposure to extreme environment such as spaceflight compared to crewmates with lower physical fitness^84^ supporting our claim that the high level of fitness in elite level athletes could explain the lack of severe symptoms.

One of the limitations of this study was that we were not able to collect saliva samples on the day of infection in the SARS-CoV-2 positive participants. While this data would have enabled us to track salivary responses to an active SARS-CoV-2 infection, it was not the purpose of the study. Also, we do not know when salivary immunity returns to normal levels, and the CDC has recently shortened the recommended period of isolation to as little as five days, which may be prior to complete recovery. Another limitation of this study is the absence of data regarding the initial viral load of the SARS-CoV-2 positive participants, since their Cycle-threshold (Ct) was not made available to the study team. Future studies should confirm the findings presented in this study by including patients with high Ct values. Finally, technical limitations associated with saliva collection led to low recovery of saliva in some participants (mostly in the SARS-CoV-2 negative group). As such, some of the analytes could not be analyzed on the entire sample population. In addition, future studies should aim to compare the recovery of immune function after infection of SARS-CoV-2 between athletes and the general population. Finally, it should be noted that the data presented in this manuscript was collected prior to SARS-CoV-2 vaccine availability, as such no participant had been vaccinated against SARS-CoV-2.

The evidence provided in the study demonstrates that the athletes enrolled in this study did not exhibit signs of salivary immunity impairments after 14-days after SARS-CoV-2 infection, suggesting that they could safely return to practice/play without a specific risk for secondary infection. Past evidence shows that high-intensity interval training has a protective rather than suppressive effect on salivary immunity in well-trained individuals^40^. Additionally, prolonged exercise and regular periodized training does not significantly change salivary IgA, while prolonged exercise increases HNP1-3 and LL-37 concentrations and secretion rates^42,43^. This is further supported by the fact that there was no difference in the rate of secondary infections or respiratory symptoms between the SARS-CoV-2 positive and negative athletes up to 3 months following their inclusion in the study. Taken together, the CDC-recommended isolation post-SARS-CoV-2 infection appears to be sufficient to normalize innate salivary immunity and lung inflammation in elite-level college athletes.

## Conclusion

The present study is the first to show that athletes who were infected with SARS-CoV-2 have a healthy salivary immune system following a period of isolation. These findings included data collected from athletes as early as 10 days post positive testing for SARS-CoV-2, and an overall average of 14 days after testing positive. These findings support the guidelines from the CDC and those followed by the NCAA that after 10 days of no symptoms you can safely end your isolation^85,86^.

## Data Availability

The data presented in this study are available on request from the corresponding author.
